# Sarcopenia as a Prognostic Marker of Early Mortality After Surgery for Acute Aortic Dissection: A Prospective Cohort Study

**DOI:** 10.3390/medsci14020177

**Published:** 2026-04-01

**Authors:** Tomasz Semań, Sabina Krupa-Nurcek, Mateusz Szczupak, Jacek Kobak, Amelia Dąbrowska, Wioletta Mędrzycka-Dąbrowska, Kazimierz Widenka

**Affiliations:** 1 Department of Surgery, Faculty of Medicine, Collegium Medicum, University of Rzeszów, 35-310 Rzeszów, Poland; tseman@ur.edu.pl (T.S.); sabinakrupa@o2.pl (S.K.-N.); kwidenka@ur.edu.pl (K.W.); 2 Department of Anesthesiology and Intensive Therapy in the Nicolaus Copernicus Hospital, 80-803 Gdańsk, Poland; szczupak.mateusz@icloud.com; 3 Department of Otolaryngology, Faculty of Medicine, Medical University of Gdańsk, Mariana Smoluchowskiego 17 Street, 80-214 Gdańsk, Poland; 4 Department of Otolaryngology, University Clinical Center, 80-210 Gdańsk, Poland; 5 Faculty of Medicine, Lazarski University in Warsaw, 02-662 Warsaw, Poland; amelia92012@gmail.com; 6 Department of Anaesthesiology Nursing & Intensive Care, Faculty of Health Sciences, Medical University of Gdansk, 80-211 Gdansk, Poland; wioletta.medrzycka-dabrowska@gumed.edu.pl

**Keywords:** sarcopenia, cardiac surgery, aortic dissection, perioperative factors, survival, complications

## Abstract

Introduction: Perioperative factors can significantly accelerate the development of sarcopenia in patients with aortic dissection, weakening their metabolic and functional reserves. Progressive sarcopenia after surgery is associated with a worse prognosis, increased mortality, and a higher risk of complications, which makes its early diagnosis and prevention key elements of care for this group of patients. Methods: The study included 116 patients hospitalized from April 2022 to May 2025 due to aortic dissection. Prospective studies were conducted using standardized tools as well as clinical data. The effect of blood transfusion, grip strength as measured with a hand dynamometer, and survival of patients after aortic dissection 3 months postoperatively were evaluated. Results: In the group of patients with a high risk of stroke, completely dependent and suffering from insomnia, transfusions were used significantly more often. SGA scores, CHA2DS2-VA score, and Barthel scale scores were dependent on the level of pain at discharge. Grip strength was significantly higher among patients who survived 3 months. The differences reached statistical significance on the second postoperative day. Conclusion: The results indicate that malnutrition is a key factor in the clinical condition of patients, increasing the risk of sarcopenia, stroke, and the severity of insomnia. At the same time, a higher degree of malnutrition is associated with reduced functional independence, which in turn affects the patient’s overall condition. The study found that malnutrition is a key factor in worsening the clinical condition of patients with aortic dissection, increasing the risk of sarcopenia, stroke and exacerbation of insomnia. The relationship between lower self-reliance and higher insomnia levels underscores the complex interplay among nutritional status, physical functioning, and sleep quality.

## 1. Introduction

Aortic dissection remains one of the most serious emergencies in vascular and cardiac surgery, characterized by high mortality in both acute and long-term periods. Despite advances in surgical techniques, anesthesiology, and intensive care, the prognosis for patients with aortic dissection still largely depends on their physiological reserve, regenerative capacity, and resistance to metabolic stress associated with surgical treatment [1]. In recent years, there has been increasing attention to sarcopenia—the progressive loss of skeletal muscle mass and function—as an important prognostic factor in patients undergoing major cardiac surgery. Sarcopenia, originally associated mainly with aging, is now understood to be a complex syndrome resulting from the interaction of inflammatory processes, metabolic disorders, malnutrition, immobilization, and oxidative stress, which can intensify in the perioperative period. In the context of aortic dissection, where surgical intervention is often urgent, extensive, and burdensome, the importance of these mechanisms may be particularly high [1,2]. Perioperative factors—including both preoperative parameters and the intra- and postoperative course—may play a key role in modulating the risk of developing or progressing sarcopenia [3]. These include nutritional status, the presence of chronic comorbidities, the degree of immune system activation, procedure duration, blood loss, the need for extracorporeal circulation, as well as the length of mechanical ventilation and hospitalization in the intensive care unit. Each of these elements can affect the balance between muscle protein synthesis and degradation, leading to muscle catabolism, which in extreme cases manifests clinically as sarcopenia [4,5]. Importantly, sarcopenia is not only a marker of overall body weakness but also actively contributes to the deterioration of treatment outcomes by increasing the risk of infectious complications, healing disorders, and respiratory failure, as well as by limiting the possibility of postoperative rehabilitation [2,3,4]. In the literature, it is increasingly emphasized that sarcopenia is an independent risk factor for increased mortality in patients undergoing cardiac surgery, including aortic repair procedures [6]. However, data on the aortic dissection population are still limited, and most available analyses focus on anatomical, technical, and hemodynamic parameters, omitting muscle reserve as a potential predictor of survival. Meanwhile, patients with aortic dissection often present numerous risk factors that promote the development of sarcopenia, such as advanced age, chronic hypertension, cardiovascular diseases, chronic inflammation, or limited physical activity before hospitalization [7]. Combined with the dramatic course of the acute phase of the disease and intensive treatment, these factors can lead to rapid deterioration of skeletal muscle in a short period of time [6,7]. Understanding the relationship between perioperative factors and the development of sarcopenia is crucial for improving treatment outcomes in patients with aortic dissection [8]. Early identification of patients at risk of losing muscle mass may enable the implementation of targeted interventions, such as optimizing nutritional status, modifying surgical strategies, reducing mechanical ventilation time, or intensifying early mobilization and rehabilitation. In addition, assessing sarcopenia—e.g., by means of computed tomography, which is routinely performed in patients with suspected aortic dissection—can be an easily accessible prognostic tool, allowing for more precise risk stratification [9]. Despite the growing interest in the topic, there is still a lack of studies comprehensively analyzing the impact of perioperative factors on the development of sarcopenia and its consequences for survival in this specific group of patients [8,10]. Previous work was often limited to single parameters, without considering the complex interaction between metabolic load, inflammatory response, oxidative stress, and catabolic processes. Meanwhile, a holistic approach that integrates clinical, imaging, and biochemical data may provide new insights into the pathophysiology of sarcopenia in the context of aortic dissection and identify potential targets for therapeutic interventions [11]. In the light of the above observations, it is necessary to deepen the understanding of the role of perioperative factors in the initiation and progression of sarcopenia and to evaluate their impact on short- and long-term survival in patients with aortic dissection. A better understanding of these relationships can not only improve prognosis but also provide a basis for developing new perioperative management strategies aimed at protecting muscle mass and function. In the era of personalized medicine, incorporating sarcopenia into risk assessment may be a key step in optimizing treatment for this highly demanding patient population.

In recent years, there has been a growing number of studies examining the importance of sarcopenia and malnutrition as risk factors for adverse outcomes in cardiovascular surgery. However, the methodology used in the works so far has been very diverse. Some studies were based on CT or DXA measurements of muscle mass, others used screening tools such as SARC-F, and still others focused solely on assessing muscle function. There is a lack of consistency in the definitions of sarcopenia, and the results regarding its effects on mortality and complications are inconclusive. Some studies indicated a strong association between low muscle strength and the risk of death after cardiac surgery, while others did not support this relationship in acute populations.

Despite the growing interest in the topic, there has been a lack of studies evaluating sarcopenia in the acute phase of aortic dissection of the type A, where the patient’s clinical condition is particularly unstable, and the time for preoperative evaluation is very limited. Previous publications have focused mainly on the anatomical and technical factors of surgery, leaving out nutritional status and muscle function as potential predictors of outcomes. This knowledge gap justifies the need for the analysis of simple, fast, and reliable tools for the assessment of sarcopenia, which can be used in the acute cardiac surgery setting.

The following research hypothesis was formulated: In patients operated on for acute aortic dissection, malnutrition and sarcopenia are significantly associated with a poorer prognosis, a higher incidence of postoperative complications, and increased mortality in the early postoperative period.

### Aim of the Study

The aim of the study was to analyze the perioperative factors in the development of sarcopenia and their impact on survival in patients with aortic dissection.

The analysis focused on identifying clinical and laboratory parameters that may predispose to loss of muscle mass and function in the perioperative period. Both factors related to the patient’s general condition before the procedure, as well as those resulting from the course of treatment and postoperative complications, were taken into account.

The study also aimed to determine the relationship between the presence or severity of sarcopenia and prognosis, including short-term and long-term mortality. Particular emphasis was placed on assessing whether sarcopenia can be an independent prognostic factor in patients with aortic dissection and whether its development can be modified by clinical interventions.

## 2. Materials and Methods

### 2.1. Study Design

The study was opportunistic (feasibility-based), and the sample size was based on the total number of eligible patients in the analyzed period. The obtained abundance provided sufficient power to detect significant differences in key analyses (e.g., differences in grip strength between patients who survived vs those who did not survive 3 months), which is confirmed by the very low *p*-values in the presented results.

The study included adult patients aged 30–77 years who were hospitalized for type A aortic dissection between 2022 and 2025.

Differences in mean values of continuous variables (e.g., pain level, grip strength) between clinical groups were analyzed. ANOVA, Kruskal–Wallis, post hoc assays, and Chiquad assay were used to assess the relationship between variables, and the results of these analyses are presented in below tables.

**Figure 1:**
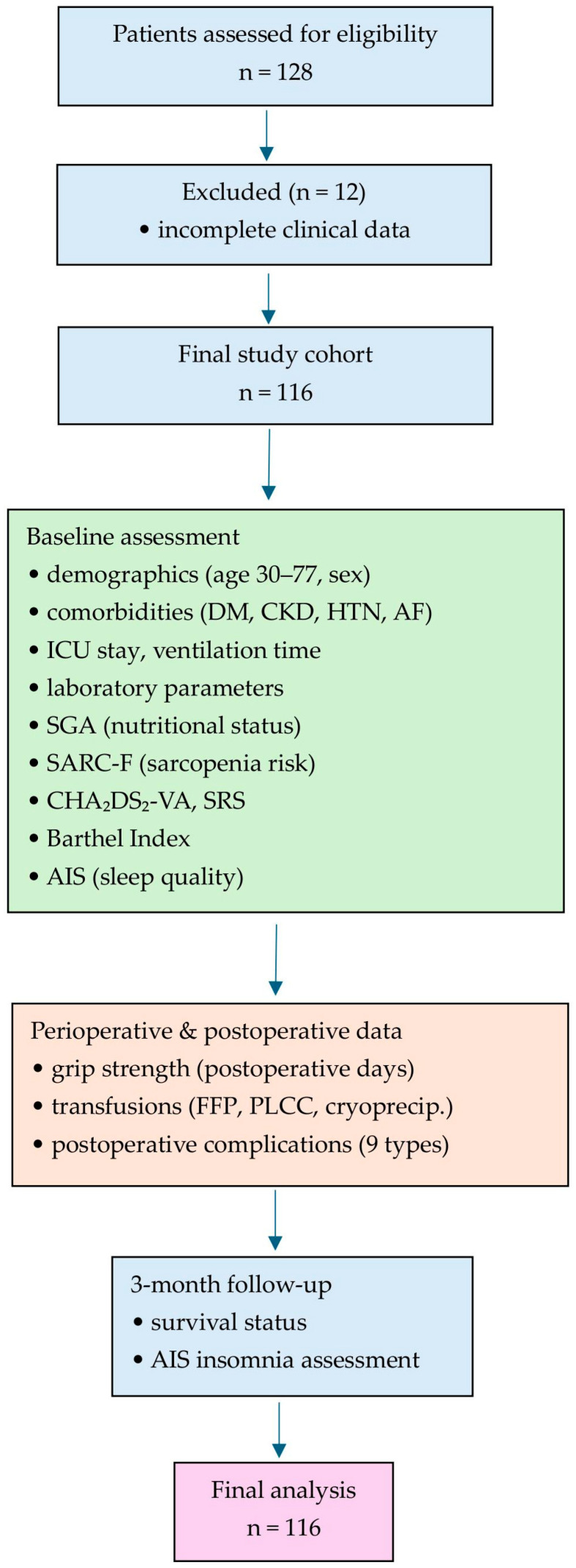
Flow diagram illustrating patient screening, inclusion, baseline assessment, perioperative data collection, and 3-month follow-up.

Figure 1 shows a flow diagram illustrating patient screening, inclusion, baseline assessment, perioperative data collection, and 3-month follow-up. Of 128 patients assessed for eligibility, 12 were excluded due to incomplete data, resulting in a final cohort of 116 individuals with type A aortic dissection.

### 2.2. Inclusion and Exclusion Criteria

- Inclusion criteria included: adult patients; informed consent to the patient’s procedure (if the patient is conscious and logical); patients with type A aortic dissection
- Exclusion criteria: underage patients; patients with type B aortic dissection; lack of consent of the patient to the procedure; lack of logical continuity.

The perioperative procedure was in accordance with the treatment protocol for acute aortic dissection type A. All patients were qualified for urgent emergency repair surgery. The standard of care included hemodynamic stabilization, blood pressure control, neurological evaluation, monitoring of laboratory parameters, and respiratory support as needed. After the operation, patients were treated in the intensive care unit, where vital signs, fluid balance, coagulation parameters, the need for blood transfusions, and the occurrence of postoperative complications were monitored. Assessment of nutritional status, risk of sarcopenia and muscle strength was an integral part of postoperative care and was performed after the clinical condition had stabilized.

### 2.3. Tools Used in the Study

SARC-F is a questionnaire used to initially assess the risk of sarcopenia, i.e., loss of muscle mass and strength. The key is as follows: 0–3 points means a low risk of sarcopenia; ≥4 points means a high risk of sarcopenia [12,13,14,15]. CHA_2_DS_2_-VA is a scale used to assess the risk of thromboembolic complications [16]. The key on this scale is as follows: 0 points—the risk of thromboembolic complications is low, so there is no need for anticoagulant therapy. After obtaining 1 point, anticoagulant therapy should be considered, depending on additional risk factors and the doctor’s decision. If a score of ≥2 points is achieved, the use of anticoagulants such as NOAC (new oral anticoagulants) or VKA (vitamin K antagonists) is recommended) [17]. Delirium was assessed using the CAM-ICU scale. The occurrence of delirium was assumed to be 1 plus 2 and either 3 or 4 = positive result [18]. Subjective global assessment (SGA) is a nutrition assessment tool that refers to an overall evaluation of a patient’s history and physical examination and uses structured clinical parameters to diagnose malnutrition [19]. Stroke risk scorecard (SRS) is a self-assessment tool that helps identify risk factors for stroke based on categories like blood pressure, smoking, cholesterol, diabetes, and exercise [20]. Barthel scale (BS) is an ordinal scale used to measure performance in activities of daily living [21]. Athens insomnia scale (AIS) is the instrument designed for quantitative measurement of the severity of insomnia based on the ICD-10 criteria [22]. The assessment of muscle function was carried out in accordance with the recommendations of the European Working Group on Sarcopenia in Older People (EWGSOP2). The protocol included a two-stage assessment: (1) sarcopenia risk screening using the SARC-F questionnaire, and (2) muscle strength measurement using a hand dynamometer. The examination was performed in the postoperative period, after the patient’s condition had stabilized, when the patient was fully conscious and able to cooperate. The patient sat with his arm bent, his elbow bent at a 90° angle, and his forearm in a neutral position. Three measurements were taken with 30 s intervals, and the highest value was taken for analysis. Hand grip force measurements were made using a calibrated hydraulic dynamometer. The calibration of the device was checked daily according to the manufacturer’s instructions. To reduce daily variability, all measurements were carried out in the morning (8:00–10:00). The patient received standardized instructions and verbal encouragement during each measurement. In the event of pain, dizziness or fatigue, the measurement was interrupted and resumed once the patient’s condition had stabilized. The thresholds for low muscle strength were EWGSOP2: <27 kg for men and <16 kg for women. The standardization of the procedure was aimed at reducing inter-individual variability and ensuring reproducibility of results.

In addition, correlations related to blood transfusions and blood products were investigated.

This study took a critical approach to assessing nutritional status and muscle function, taking into account current EWGSOP2 recommendations. Hand grip strength was measured using a dynamometer as a primary marker of muscle function. The choice of this method resulted from its high reliability, availability and the possibility of using it in the acute phase of aortic dissection, in which imaging methods are often not feasible. Statistical analyses included cross-clinical comparisons, assessment of postoperative complications, and survival analysis, according to previously defined dependent and independent variables.

### 2.4. Reference Intervals of Test Results

In the case of obesity, it was assumed that the BMI was 30 and more. Pain, in turn, was assessed on the NRS scale, where 0 means no pain, 1–4 is mild pain, 5–6 is moderate pain, 7–10 is severe pain. The strength of the grip measured with a dynamometer was dependent on the sex and age of the patients and readable from the table [23].

### 2.5. Statistical Methods

In order to examine the distribution of the results of the variable age, duration of treatment, length of stay, ventilation time, pain level, troponin, protein, urea, and creatinine, the Shapiro–Wilk test was used (W).

In order to investigate the distribution of the results of dependent variables in groups of independent variables, the chi-square (χ2) test was used. To compare the means of the tested variables with a normal distribution across groups, the ANOVA (F) test was used. In the ANOVA analysis, η^2^ was added to explain the variability of the dependent variable and post hoc NIR testing. In order to compare the average scores of the tested variables that did not meet the criterion of the normal distribution in the groups, the Kruskal–Wallis ANOVA test was used, and a control of multiple rank comparisons for all groups was added. The significance level was *p* < 0.05. To check the correlation of variable scales, the correlation of the Spearman R. scales was performed at *p* < 0.05.

#### 2.5.1. The Potential Role of Multivariate Analysis

The study assessed numerous clinical, laboratory, and functional factors that may influence both the development of sarcopenia and survival after aortic dissection surgery. Variables with documented significance included age, comorbidities (e.g., diabetes, chronic kidney disease, hypertension), ventilation time, length of stay in the intensive care unit, and nutritional status. The text emphasized that “malnutrition is a key factor in the clinical condition of patients, increasing the risk of sarcopenia, stroke, and the severity of insomnia,” and that “grip strength was significantly higher among patients who survived 3 months.”

Incorporating multivariate analysis (e.g., logistic or Cox regression) would allow us to assess whether sarcopenia is an independent predictor of mortality after adjusting for confounding variables. This approach would allow us to separate the impact of sarcopenia itself from the effects of age, medical conditions, or the severity of the postoperative course. In the context of the observed relationships—e.g., the higher incidence of complications in patients at high risk of sarcopenia (“wound infection, tracheostomy, bedside dialysis, delirium, bedsores, SCA, and other complications were significantly more common”)—multivariate analysis could indicate which of these relationships are independent and which result from overlapping clinical factors.

#### 2.5.2. Approach to Multiple Comparisons

The study conducted a large number of statistical tests, including between-group comparisons based on SGA, SARC-F, CHA2DS2-VA, Barthel Index, AIS, and numerous postoperative complications. Chi-square tests, ANOVA, Kruskal–Wallis test, and multiple rank comparisons were used. The text indicates that “a control of multiple rank comparisons for all groups was added,” meaning that the authors included correction for multiple comparisons in nonparametric analyses.

In the context of the number of tests performed, it is important to reduce the risk of Type I error. Using procedures such as Bonferroni correction, Holm–Bonferroni correction, or FDR control could further enhance the reliability of the results, especially in analyses involving multiple categories (e.g., three levels of SGA, three levels of AIS, three levels of Barthel Index). In this study, results with very low *p* values were particularly significant, e.g., “*p* = 0.000001” for the association between SGA and transfusions, indicating strong effects that are resistant to multiple adjustments.

#### 2.5.3. Definition of Sarcopenia According to Guidelines European Working Group on Sarcopenia in Older People 2 (EWGSOP2)

Sarcopenia was defined according to the guidelines of the European Working Group on Sarcopenia in Older People 2 (EWGSOP2). The EWGSOP2 diagnostic model is hierarchical and based on three levels of assessment:

- Reduced muscle strength—the basic and most sensitive criterion for recognizing probable sarcopenia.
- Decreased muscle mass—necessary to confirm sarcopenia.
- Decreased physical performance—indicates severe sarcopenia (severe sarcopenia).

The study used hand grip strength as the main functional criterion, in accordance with the recommendations of EWGSOP2. Measurements were taken with a calibrated hydraulic dynamometer, and the limit values for low muscle strength were assumed to be <27 kg for men and <16 kg for women.

Due to the sudden nature of acute aortic dissection of the type A and the need for urgent surgical intervention, it was not possible to perform measurements of muscle mass using imaging methods (CT, MRI, DXA, BIA) in the preoperative period or in the early postoperative period. For this reason, in accordance with the recommendations of EWGSOP2 for clinical situations in which the assessment of muscle mass is not feasible, sarcopenia was operationalized as probable sarcopenia, based solely on the criterion of low muscle strength. In addition, the SARC-F questionnaire was used as a screening tool to assess the risk of sarcopenia (≥4 points = high risk), however, the diagnosis of sarcopenia was based solely on the criterion of muscle strength according to EWGSOP2 and not on the SARC-F score.

### 2.6. Ethical Statement

An application was submitted to the Bioethics Committee at the University of Rzeszów (KBE No. 2022/058) to obtain a positive opinion on the study.

## 3. Results

### 3.1. Participants

The study included patients scheduled for cardiac surgery. The study ran from April 2022 to the end of May 2025. Data from a total of 128 patients were analyzed, of whom 12 were excluded (not all data were available), ultimately enrolling 116 individuals.

There were 44 women (37.93%) and 72 men (62.07%) in the study group. The students’ ages ranged from 30 to 77 years (x = 57.99 ± 8.5, ME = 58.5, *p* = 0.08). In the study group, comorbidities were analyzed: diabetes occurred in 33 participants (28.45%), obesity in 16 participants (13.79%), and hypertension in 46 participants (39.66%); 58 people (50%) had atrial fibrillation and 39 patients (33.62%) suffered from chronic kidney disease. The duration of stay in the Intensive Care Unit (ICU) after surgery ranged from 49 to 1321 h (x = 306.84 ± 395.63, ME = 143, *p* < 0.000001). The ventilation time was carried out from 115 to 1445 min (x = 762.41 ± 314.59, ME = 752.5, *p* < 0.000001). Nicotine was present in 58 patients (50%). The results of the research are presented in Table 1.

### 3.2. Parameters Related to Rolls in Groups of Dependent Variables

**Figure 2:**
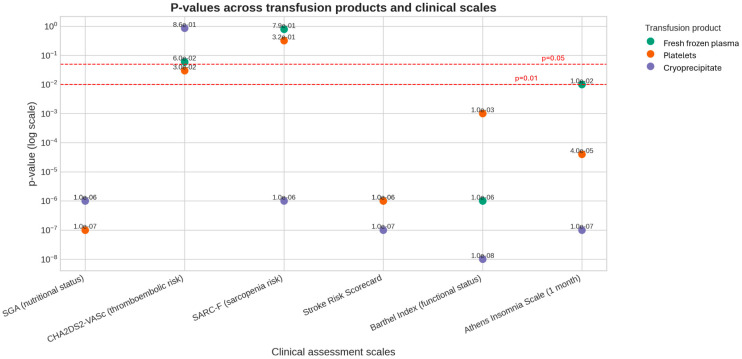
*p*-values across transfusion products and clinical scales.

The distribution of parameters related to transfusions of blood products in groups of variables dependent on the chi-square test was checked. In the group of severely malnourished patients, freshly frozen plasma (FFP) and platelet blood cell concentrate (PLCC) were transfused significantly more often and cryoprecipitate transfusion was performed. Among patients with a CHA_2_DS_2_-VA score of 1, PLCC was more frequently transfused. In the group of patients with a high risk of sarcopenia, cryoprecipitate transfusion was performed more frequently. In the group of patients with a high risk of stroke, who were completely dependent and suffering from insomnia, FFP and PLCC transfusions were significantly more frequent, and cryoprecipitate transfusions were performed. The results are presented in Table 2 and graphically in Figure 2.

### 3.3. Complications in Groups of Dependent Variables

In order to describe the complications occurring in patients, nine complications were included in the group of complications: pressure ulcers (1), pain (2), SCA (3,4), wound infection (5), tracheostomy (6), dialysis (7), other complications (8), and delirium (9). Among the other complications, chest revision was noted.

#### 3.3.1. Pain

In the study group, pain was reported by all patients (min = 3, max = 6), with an average of 4.30 ± 1.08 and a median ME = 4. Mean pain levels were compared in groups of dependent variables using the ANOVA(F) analysis of variance. SGA scores, CHA_2_DS_2_-VA score, and Barthel scale scores were found to be dependent on the variability in discharge pain levels. The results are presented in Table 3 and graphically in Figure 2.

#### 3.3.2. Subjective Global Assessment 

The level of pain at discharge from the ward was significantly higher than the level of pain at discharge of patients in the group of mildly and moderately malnourished patients.

#### 3.3.3. CHA_2_DS_2_-VA Results

The level of pain at discharge from the ward in patients with a higher risk of thromboembolic complications was significantly higher than the level of pain at discharge of other patients.

#### 3.3.4. Barthel Scale

The level of pain at discharge from the ward in completely dependent patients was significantly higher than the level of pain at discharge of partially dependent patients and in fully independent patients.

**Figure 3:**
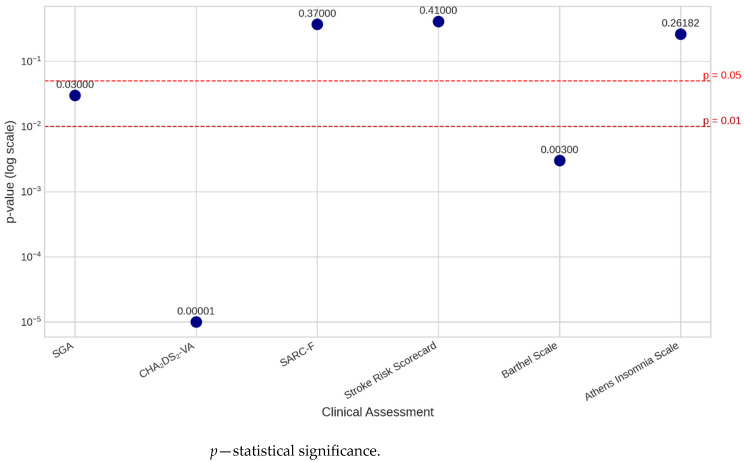
*p*-values across clinical assessments.

Below in graphic form (Figure 3), *p*-values across clinical assessments are presented. Writing in the form aeb means a⋅10b, which is the number a multiplied by 10 raised to the power of b.

### 3.4. Other Postoperative Complications

The distribution of complications in groups of dependent variables was checked using the chi-square test. The results are presented in Table 4 and graphically in Figure 3.

#### 3.4.1. Subjective Global Assessment 

In the severely malnourished group, wound infection, tracheostomy, delirium, pressure ulcers, sudden cardiac arrest (SCA), and other complications occurred.

#### 3.4.2. CHA_2_DS_2_-VA Results

Pressure ulcers were significantly more common in patients with a CHA_2_DS_2_-VA score of 2 or higher.

#### 3.4.3. SARC-F

In the group of patients with a high risk of sarcopenia, wound infection, tracheostomy, bedside dialysis, delirium, bedsores, SCA, and other complications were significantly more common.

#### 3.4.4. Stroke Risk Scorecard

Wound infection, tracheostomy, bedside dialysis, delirium, pressure ulcers, SCA, and other complications were more common in the group of patients at high risk of stroke.

#### 3.4.5. Barthel Scale

In the group of completely dependent patients, wound infection, tracheostomy, other complications and delirium were more common.

#### 3.4.6. Athens Insomnia Scale

In the group of patients with insomnia, wound infection, tracheostomy, bedside dialysis, other complications, and delirium were more common.

Complications were treated as binary variables (occurred/did not occur), with no severity gradation. The list of complications included wound infection, tracheostomy, postoperative dialysis, delirium, pressure ulcers, sudden cardiac arrest (SCA), pain, other complications (e.g., chest revision).

**Figure 4:**
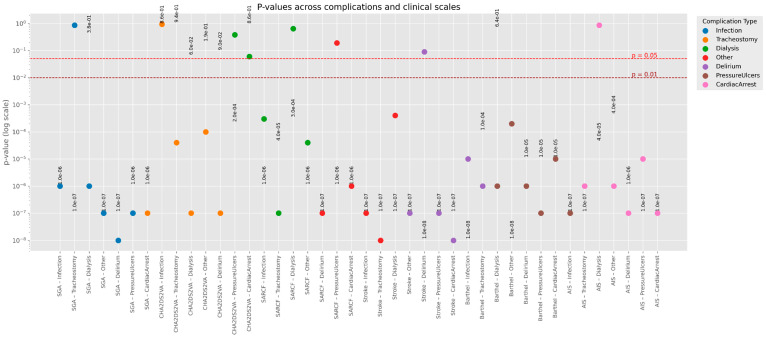
*p*-values across complications and clinical scales. *p*—statistical significance. Legend: aeb represents the number: a × 10^b^, that is, the number a multiplied by 10 raised to the power of b.

Figure 4 graphically shows *p*-values across complications and clinical scales. Writing in the form aeb means a⋅10b, which is the number a multiplied by 10 raised to the power of b. 

### 3.5. Months Survival

The distribution of survival 3 months post-treatment in groups of chi-squared dependent variables was checked. The results are presented in Table 5 and graphically in Figure 4.

Subjective Global Assessment

In the group of severely malnourished patients, 3-month survival occurred significantly less frequently than in the other groups.

CHA_2_DS_2_-VA results

Among patients with scores above 2, 3-month survival occurred significantly less often than among patients with a score of 1 (with a lower risk of thromboembolic complications).

SARC-F

In the group of patients at high risk of sarcopenia, survival of 3 months was significantly less common than in the group of patients with a low risk of sarcopenia.

Stroke Risk Scorecard

In the group of patients with a high risk of stroke, survival of 3 months was significantly less common than in the group of patients with a moderate to low risk of stroke.

Skala Barthel

Among patients with complete dependence, 3-month survival occurred significantly less frequently than among patients who were moderately or completely independent.

Athens Insomnia Scale

In the group of patients with insomnia, survival of 3 months occurred significantly less often than in the group of patients with insomnia bordering on the norm or without insomnia.

**Figure 5:**
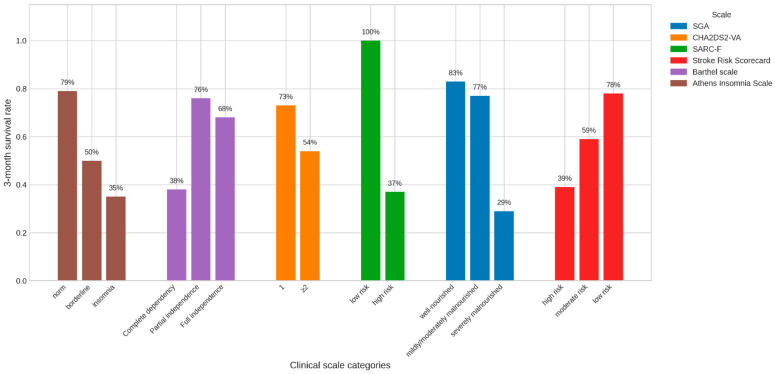
Three-month survival across clinical scales.

Below is a graphic presentation of 3-month survival across clinical scales (Figure 5).

### 3.6. Grip Strength in Groups of Dependent Variables

The distribution of grip force across the dependent variables was examined. The results are presented in Table 6.

#### 3.6.1. Subjective Global Assessment 

The statistical significance of the nutritional status of patients in all groups (well-nourished, mildly/moderately malnourished, severely malnourished) in relation to the strength of the grip on days 5 and 7 was demonstrated.

#### 3.6.2. SARC-F

A statistical relationship between the risk of sarcopenia (both low and high risk) and the strength of the patient’s grip on days 2, 5, and 7 was demonstrated.

#### 3.6.3. Athens Insomnia Scale

In the group of patients with insomnia, survival of 3 months occurred significantly less often than in the group of patients with insomnia bordering on the norm or without insomnia.

In the case of the thromboembolic complications risk scale, stroke risk scale, and the Barthel scale, no statistical significance was recorded in relation to grip strength at days 2, 5, 7, and 10.

### 3.7. Grip Strength, Complications

The distribution of postoperative complications in the postoperative days: second, fifth, seventh, and tenth was checked. The results are presented in Table 7.

The most pronounced differences in grip strength were observed in patients with wound infection, pressure ulcers, delirium, and after sudden cardiac arrest. In these groups, grip strength was significantly lower than in patients without complications, particularly from the 5th postoperative day onward. For wound infections, significant differences were noted in day 5 (*p* = 0.005), day 7 (*p* = 0.01), and day 10 (*p* = 0.02), with effect magnitudes indicating moderate dependence strength (η^2^ 0.04–0.07). A similar pattern was observed for pressure ulcers (*p* = 0.005 and *p* = 0.01 on day 5 and day 7, respectively). Delusions were associated with significantly lower grip strength on days 5 (*p* = 0.01) and days 7 (*p* = 0.03), while patients after sudden cardiac arrest showed a significant decrease in grip strength on days 5, 7, and 10 (*p* = 0.005, 0.01, 0.02).

For tracheostomy, bedside dialysis and other complications, only single significant differences were observed, mainly on day 5 or 7, with small values of η^2^ (≤0.07), suggesting limited clinical relevance of these relationships. The absence of significant differences on Day 2 for all analyzed complications indicates that early postoperative grip strength does not yet reflect the impact of developing complications.

Analysis of the cumulative number of complications showed that patients with one complication exhibited significantly higher grip force than those with three or more complications, particularly on days 5 (*p* = 0.01) and 7 (*p* = 0.04).

### 3.8. Grip Strength, Survival After 3 Months

The table shows the mean handshake strength (x) and standard deviation (SD) on the 2nd, 5th, 7th, and 10th postoperative days, depending on the 3-month survival of the patients. Analysis of variance showed significant differences between patients who survived for 3 months and those who died during this period. Table 8 shows the results.

At all time points, grip strength was markedly higher in patients who survived for 3 months. The differences reached statistical significance as early as day 2 (F = 4.85; *p* = 0.03; η^2^ = 0.05) and then persisted on day 5 (F = 7.27; *p* = 0.01; η^2^ = 0.06) and day 7 (F = 4.86; *p* = 0.03; η^2^ = 0.04). On day 10, a trend towards significance was observed (F = 3.53; *p* = 0.06), which may be due to greater variability in measurements later in the recovery period.

Effect sizes (η^2^ = 0.04–0.06) indicate a small-to-moderate effect of survival on grip strength, consistent with clinical observations that muscle strength is a sensitive marker of the patient’s overall condition and prognosis. Patients who did not survive for 3 months had consistently lower grip strength on all days, suggesting that muscle weakness may be an early sign of deterioration in general condition and increased risk of death.

**Figure 6:**
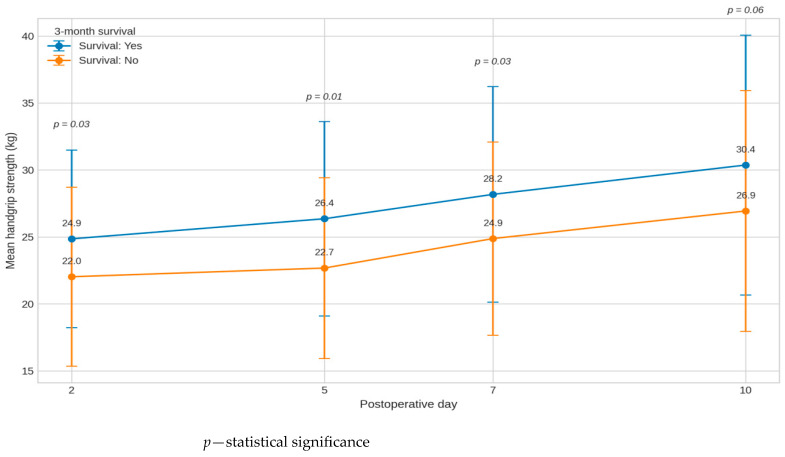
Handgrip strength across postoperative days by 3-month survival status.

The graph (Figure 6) presents handgrip strength across postoperative days by 3-month survival status. The graph shows the standard deviation and *p*-values for each pair of points on the day under study (2, 5, 7, and 10).

### 3.9. The Primary Endpoint and the Possible Secondary Outcomes

#### 3.9.1. Primary Endpoint

The primary endpoint was early postoperative mortality (90-day survival) in patients undergoing surgery for acute aortic dissection, assessed in relation to the presence and severity of sarcopenia. The assessment included the relationship between handgrip strength, risk of sarcopenia (SARC-F), nutritional status (SGA), and postoperative survival. This was based on the study’s assumption that grip strength was significantly higher among patients who survived 3 months and the aim was to analyze the perioperative factors in the development of sarcopenia and their impact on survival in patients with aortic dissection.

#### 3.9.2. Secondary Outcomes

Incidence and progression of sarcopenia in the perioperative period, assessed by SARC-F and measurement of hand grip strength. This section examines the association between nutritional status and clinical outcomes, including the relationship between SGA and complications, pain at discharge, and functional performance. Use of blood products (FFP, PLCC, cryoprecipitate) depended on nutritional status, risk of sarcopenia, risk of stroke, level of independence and quality of sleep. Incidence of postoperative complications were wound infection, tracheostomy, dialysis, delirium, pressure ulcers, sudden cardiac arrest, and other complications. Functional status at discharge was assessed by the Barthel Index. Sleep quality one month after discharge was assessed by the Athens Insomnia Scale. Perioperative clinical and laboratory parameters including ventilation time, length of stay in the ICU, troponin, protein, creatinine, urea, potentially modulated the risk of sarcopenia.

## 4. Discussion

The results of the study confirm that perioperative factors play a key role in the development of sarcopenia and in the prognosis of patients with aortic dissection type A. These relationships are increasingly described in the international literature, indicating that patients undergoing urgent cardiac surgery are at particularly high risk of rapid postoperative loss of muscle mass and function due to metabolic stress, inflammatory responses, and immobilization [12,24]. Our results are consistent with these observations, confirming that both nutritional status and perioperative clinical parameters significantly affect the development of sarcopenia and patient survival.

One of the most important findings of the study is the strong association between malnutrition as assessed by SGA and the frequency of blood product transfusions. Severely malnourished patients required more frequent transfusions of FFP, PLCC, and cryoprecipitate, which may indicate poorer hemostasis and greater susceptibility to coagulation disorders. Similar results were reported by Cruz-Jentoft et al., who showed that malnutrition is an independent predictor of increased transfusion requirements in patients undergoing cardiac surgery. In turn, Krupa et al. emphasized that malnutrition intensifies muscle catabolism, thereby accelerating the progression of sarcopenia during the perioperative period [25].

In our study, patients at high risk of sarcopenia (SARC-F ≥ 4) were more likely to experience complications such as wound infection, tracheostomy, dialysis, delirium, pressure ulcers, and sudden cardiac arrest. These results are consistent with the observations of Lee et al., who showed that sarcopenia is a strong predictor of infectious and respiratory complications after cardiac surgery [26]. Similarly, Wang et al. [27] indicated that reduced muscle mass is associated with increased susceptibility to infections and prolonged hospitalization. These mechanisms may result from weakened immune function, impaired tissue regeneration, and reduced metabolic reserve, as confirmed by Johnson et al. [28].

An important element of our study is the analysis of grip strength as an objective indicator of muscle function. Patients who survived 3 months postoperatively had significantly higher grip strength on the second postoperative day. This result is consistent with the reports of Santos et al., who indicated that grip strength is one of the most sensitive predictors of postoperative mortality. In turn, Patel et al. showed that low grip force correlates with longer mechanical ventilation and a higher risk of respiratory complications after aortic surgery [29]. The relationship is worth noting between the level of pain at discharge and the results of the SGA, CHA_2_DS_2_-VA, and Barthel scales. Malnourished patients, at higher risk of thromboembolic complications and completely functionally dependent, reported higher levels of pain. Similar observations were reported by Martínez-Arnau et al., who indicated that malnutrition and sarcopenia may exacerbate pain perception through increased inflammatory activation [30]. In turn, Tazreean et al. emphasized that patients with limited postoperative mobility are more likely to experience chronic pain, which may further complicate rehabilitation [31]. Hand grip strength is recommended by EWGSOP2 as a basic marker of muscle function and a key element in the diagnosis of sarcopenia. Unlike imaging methods such as CT or DXA, the measurement of grip force is fast, non-invasive, and possible even in acute patients. In cardiac and cardiac surgery populations, low grip strength has been shown to be an independent predictor of mortality, postoperative complications, and prolonged hospitalization time. For this reason, dynamometry is the most practical tool for assessing muscle function in patients with acute aortic dissection, for whom time and safety of diagnostic procedures are crucial.

Results on postoperative complications are particularly important in the care of patients with aortic dissection. In our analysis, patients with a high risk of sarcopenia, a high risk of stroke, and low functional independence were more likely to experience wound infections, delirium, pressure sores, tracheostomy, and sudden cardiac arrest. Similar relationships were described by O’Connor et al., who showed that sarcopenia is an independent predictor of respiratory complications, sepsis, and 90-day mortality in the intensive care population [32]. In turn, Harris et al. indicated that patients with low muscle mass are more likely to develop delirium, which may result from metabolic disorders, hypoxia, and increased oxidative stress [33].

It is also worth emphasizing the role of insomnia assessed with AIS. Patients with insomnia were more likely to experience complications such as wound infection, dialysis, delirium, or tracheostomy. Sleep disorders are increasingly recognized as a significant risk factor for deterioration of immune function and increased inflammatory response, which can accelerate the development of sarcopenia. Research conducted by Rampes et al. indicated that postoperative insomnia is associated with a higher risk of delirium, as well as a worse long-term prognosis [34]. Similar results were presented by Carter et al., who showed that sleep disorders affect muscle protein metabolism and increase the risk of muscle loss [35].

The results of our study fit into the growing body of literature indicating the importance of sarcopenia and malnutrition as risk factors for adverse outcomes in cardiovascular surgery. At the same time, they confirm that the data so far are ambiguous, which results from differences in the methodology of previous research. In contrast to work based on muscle mass measurements, our study focused on muscle function, which is in line with current EWGSOP2 recommendations. The results confirm that grip strength is a sensitive risk marker, and its reduction is associated with a higher incidence of complications and lower survival. At the same time, our data fill a gap in the literature, as previous studies have not analyzed sarcopenia in the context of acute aortic dissection type A. In this population, the use of rapid and emergency-capable tools is particularly important, further reinforcing the use of dynamometry as a key element of patient assessment.

## 5. Conclusions

The study provides important data confirming the key role of perioperative factors in the development of sarcopenia and their impact on the survival of patients with aortic dissection. These results highlight the need to implement an integrated approach to the care of this group of patients, including optimization of nutritional status, monitoring of muscle function, early mobilization, and prevention of complications. In the future, multicenter studies are advisable to confirm the results obtained and to develop standards of management aimed at reducing the risk of sarcopenia in the population of patients with aortic dissection. Table 9 is provided below to summarize the key perioperative factors associated with the risk of developing sarcopenia

## 6. Study Limitations

Although this study makes an important contribution to understanding the role of perioperative factors in the development of sarcopenia in patients with type A aortic dissection, it has several limitations that should be considered when interpreting the results. First, it is a single-center study, which limits the possibility of generalizing the results to populations with different demographic, organizational, or therapeutic profiles. Clinical practices, transfusion protocols, nutritional strategies, and standards of postoperative care may vary across centers, potentially affecting the incidence of complications and the dynamics of sarcopenia development. Second, although the study was prospective, some of the data were based on subjective scales (e.g., SARC-F, AIS, SGA), which may be prone to declarative bias, especially in patients who have undergone major cardiac surgery and who experience impaired consciousness, delirium, or fatigue. This can affect the accuracy of the assessment of the risk of sarcopenia or the quality of sleep. Third, the assessment of grip strength was performed in a clinical setting, in a short postoperative period, which could be impaired by pain, sedation, fatigue, or movement limitations. Although grip strength is a recognized marker of muscle function, its measurement at such an early postoperative period may not fully reflect the patient’s actual muscle reserve. Fourth, the study did not use imaging methods for assessing muscle mass (e.g., CT L3, muscle ultrasound), which are currently considered the gold standard in the diagnosis of sarcopenia. The use of only clinical and functional tools limits the possibility of a complete characterization of the sarcopenia phenotype. Fifth, the analysis of postoperative complications covered a wide spectrum of events but did not account for their severity or time of onset. The lack of stratification of complications may limit the possibility of precisely determining their causal relationship with malnutrition, sarcopenia, or other perioperative factors. Sixth, the follow-up period was 3 months, which allows for an assessment of early mortality, but does not allow for an analysis of the long-term consequences of sarcopenia, such as permanent disability, rehospitalization, or annual mortality. In the case of aortic dissection, a disease with a high risk of distant complications, longer follow-up could yield more comprehensive data. Finally, although numerous clinical factors were considered in the analysis, no multivariate analysis was performed to identify independent predictors of the development of sarcopenia and survival. The use of regression models could increase inferential power and enable a more precise assessment of the impact of individual variables.

The limitation of the examination was in the way postoperative complications were defined and recorded. Adverse events were presented as a simple list of nine possible complications, such as wound infection, tracheostomy, dialysis, delirium, pressure ulcers, sudden cardiac arrest, pain, and other complications. As indicated in the text, “nine complications were included in the group of complications: pressure ulcers, pain, SCA, wound infection, tracheostomy, dialysis, other complications, and delirium.” Complications were therefore treated exclusively in binary terms—as present or absent—without the use of any standardized classification of their severity.

In the conditions of acute cardiac surgery, it was only possible to diagnose “probable sarcopenia”, and the lack of muscle mass assessment was due to clinical and logistical constraints.

## 7. Implications for Practice

The study’s results are of significant practical importance and indicate the need to implement a more integrated approach to the care of patients with type A aortic dissection. First, the established association between malnutrition, the risk of sarcopenia, and the incidence of postoperative complications highlights the need for routine assessment of nutritional status at the time of patient admission. Instruments such as SGA or SARC-F can be used quickly and at no additional cost, making them useful in acute cardiac surgery settings. Second, the results indicate that patients at high risk of sarcopenia should be included in early nutritional intervention, preferably before surgery, if clinical conditions allow. In practice, this may include a dietary consultation, protein supplementation, assessment of energy requirements, and monitoring of metabolic parameters. Third, the important role of grip strength as a predictor of survival suggests that this measurement can be used as a rapid indicator of risk in the postoperative period. Regular evaluation of muscle function can help identify patients who require more intensive rehabilitation or nutritional support. Fourth, the observed association between insomnia and postoperative complications underscores the need to monitor sleep quality and implement non-pharmacological interventions (e.g., sleep hygiene, environmental optimization, and noise and light reduction in the ICU). Sleep disorders can exacerbate inflammation, increase the risk of delirium, and impair muscle recovery, so their early detection is clinically important. Fifth, patients at high risk of stroke, completely functionally dependent, or severely malnourished should be treated as a special risk group, requiring more intensive monitoring, more frequent evaluation of complications, and a more aggressive rehabilitation strategy. Sixth, the results of the study highlight the need for a multidisciplinary approach, involving the collaboration of cardiac surgeons, anesthesiologists, dieticians, physiotherapists, intensive care nurses, and sleep medicine specialists. Only this approach can effectively limit the development of sarcopenia and improve the prognosis of patients. Finally, the data obtained indicate that the inclusion of sarcopenia assessment in standard perioperative protocols may represent a new element of risk stratification that will allow for more precise care planning and optimization of treatment outcomes.

## Figures and Tables

**Table 1:** Indicators of independent variables and distributions of continuous variables.

Indicators	N	%
age	x = 57.99 ± 8.5, ME = 58.5, 30–77 Shapiro–Wilk W = 0.98, *p* = 0.08	
55 years and less	44	37.93%
56–65 years	52	44.83%
66 years and more	20	17.24%
**gender**		
woman	44	37.93%
man	72	62.07%
**diabetes**		
yes	33	28.45%
no	83	71.55%
**obesity**		
yes	16	13.79%
no	100	86.21%
**hypertension**		
yes	46	39.66%
no	70	60.34%
**FA—atrial fibrillation**		
yes	58	50%
no	58	50%
**chronic kidney disease**		
yes	39	33.62%
no	77	66.38%
**duration of stay in ICU [h]**	**x = 306.84 ± 395.63, ME = 143, 49–1321** **Shapiro–Wilk W = 0.57, *p* < 0.000001**	
**ventilation time [min]**	**x = 762.41 ± 314.59, ME = 752.5, 115–1445** **Shapiro–Wilk W = 0.91, *p* < 0.000001**	
**nicotine**		
yes	58	50%
no	58	50%

x—average; ME—median; W—test statistics; *p*—statistical significance.

**Table 2:** Distribution of parameters related to rolls in groups of dependent variables.

	FFP						PLCC						Cryoprecypitate						Total
	Yes		No		chi	*p*	Yes		No		chi	*p*	Yes		No		chi	*p*	N
	N	%	N	%			N	%	N	%			N	%	N	%			
**SGA—Subjective Global Assessment**																			
well-nourished	20	42%	28	58%	51.19	0.000001	10	21%	38	79%	28.65	0.0000001	0	0%	48	100%	50.77	0.000001	48
mildly/moderately malnourished	0	0%	30	100%			0	0%	30	100%			0	0%	30	100%			30
severely malnourished	29	76%	9	24%			19	50%	19	50%			19	50%	19	50%			38
**CHA_2_DS_2_-VA—assessment of the risk of thromboembolic complications in patients after surgery**																			
1	20	34%	39	66%	3.44	0.06	20	34%	39	66%	5.17	0.03	10	17%	49	83%	0.02	0.86	59
≥2	29	51%	28	49%			9	16%	48	84%			9	16%	48	84%			57
**SARC-F—assessment of sarcopenia risk after discharge**																			
low risk of sarcopenia	20	41%	29	59%	0.07	0.79	10	20%	39	80%	0.96	0.32	0	0%	49	100%	23.54	0.000001	49
high risk of sarcopenia	29	43%	38	57%			19	28%	48	72%			19	28%	48	72%			67
**Stroke Risk Scorecard**																			
high risk	19	68%	9	32%	33.21	0.000001	19	68%	9	32%	57.93	0.000001	19	68%	9	32%	68.28	0.0000001	28
moderate risk	20	69%	9	31%			10	34%	19	66%			0	0%	29	100%			29
low risk	10	17%	49	83%			0	0%	59	100%			0	0%	59	100%			59
**Bathel Scale—assessment of the patient’s independence and fitness**																			
complete dependency	22	85%	4	15%	25.9	0.000001	14	54%	12	46%	13.48	0.001	14	54%	12	46%	28.94	0.00000001	26
partial independence	12	32%	25	68%			6	16%	31	84%			2	5%	35	95%			37
full independence	15	28%	38	72%			9	17%	44	83%			3	6%	50	94%			53
**Athens Insomnia Scale—one month after discharge**																			
norm	29	41%	41	59%	8.36	0.01	10	14%	60	86%	21.49	0.00004	0	0%	70	100%	51.89	0.0000001	70
borderline of the norm	4	20%	16	80%			3	15%	17	85%			3	15%	17	85%			20
insomnia	16	62%	10	38%			16	62%	10	38%			16	62%	10	38%			26
Total	49	42%	67	58%			29	25%	87	75%			19	16%	97	84%			116

*p*—statistical significance; N—numbers; chi—chi-square test.

**Table 3:** Significance of mean pain level at discharge in groups of dependent variables.

	Pain Level at Discharge from the Ward				
	x	SD	F	*p*	η^2^
**SGA—Subjective Global Assessment**					
well-nourished	4.21	1.01	3.56	0.03 C > B	0.05
mildly/moderately malnourished	4.00	0.00			
severely malnourished	4.66	1.46			
**CHA_2_DS_2_-VA—assessment of the risk of thromboembolic complications in patients after surgery**					
1	3.90	0.40	19.39	0.00001 B > A	0.14
≥2	4.72	1.37			
**SARC-F—assessment of sarcopenia risk after discharge**					
low risk of sarcopenia	4.41	0.81	0.82	0.37	
high risk of sarcopenia	4.22	1.24			
**Stroke Risk Scorecard**					
high risk	4.43	1.23	0.90	0.41	
moderate risk	4.45	1.24			
low risk	4.17	0.91			
**Bathel Scale—assessment of the patient’s independence and fitness**					
complete dependency	4.92	1.29	6.07	0.003 A > B, A > C	0.10
partial independence	4.16	0.99			
full independence	4.09	0.93			
**Athens Insomnia Scale—one month after discharge**					
norm	4.40		1.35612	0.261820	
borderline of the norm	3.95	0.60			
insomnia	4.31	1.41			

*p*—statistical significance; x—average; SD—standard deviation; η^2^—measure of effect size.

**Table 4:** Distribution of complications in the group of dependent variables.

	Wound Infection						Tracheostomy						Dialysis after the Procedure						Other Complications						Delirium						Pressure Ulcers						Sudden Cardiac Arrest						Total
	Yes		No		chi	*p*	Yes		No		chi	*p*	Yes		No		chi	*p*	Yes		No		chi	*p*	Yes		No		chi	*p*	Yes		No		chi	*p*	Yes		No		chi	*p*	N
	N	%	N	%			N	%	N	%			N	%	N	%			N	%	N	%			N	%	N	%			N	%	N	%			N	%	N	%			
**SGA—Subjective Global Assessment**																																											
well-nourished	0	0%	48	100%	50.77	0.000001	0	0%	48	100%	35.42	0.0000001	28	58%	20	42%	1.93	0.38	0	0%	48	100%	57.46	0.0000001	0	0%	48	100%	77.43	0.0000001	0	0%	48	100%	146.72	0.000001	0	0%	48	100%	50.77	0.000001	48
mildly/moderately malnourished	0	0%	30	100%			0	0%	30	100%			20	67%	10	33%			0	0%	30	100%			10	33%	20	67%			0	0%	30	100%			0	0%	30	100%			30
severely malnourished	19	50%	19	50%			14	37%	24	63%			19	50%	19	50%			21	55%	17	45%			38	100%	0	0%			38	100%	0	0%			19	50%	19	50%			38
**CHA_2_DS_2_-VA—assessment of the risk of thromboembolic complications in patients after surgery**																																											
1	10	17%	49	83%	0.02	0.86	7	12%	52	88%	0.004	0.94	39	66%	20	34%	3.44	0.06	8	14%	51	86%	1.68	0.19	20	34%	39	66%	2.78	0.09	10	16.95%	49	83.05%	14.02	0.0002	10	17%	49	83%	0.02	0.86	59
≥2	9	16%	48	84%			7	12%	50	88%			28	49%	29	51%			13	23%	44	77%			28	49%	29	51%			28	49.12%	29	50.88%			9	16%	48	84%			57
**SARC-F—assessment of sarcopenia risk after discharge**																																											
low risk of sarcopenia	0	0%	49	100%	23.54	0.000001	0	0%	49	100%	16.76	0.00004	19	39%	30	61%	12.66	0.0003	0	0%	49	100%	26.4	0.000001	0	0%	49	100%	77.43	0.0000001	0	0.00%	49	100.00%	55.06	0.000001	0	0%	49	100%	23.54	0.000001	49
high risk of sarcopenia	19	28%	48	72%			14	21%	53	79%			48	72%	19	28%			21	31%	46	69%			48	72%	19	28%			38	56.72%	29	43.28%			19	28%	48	72%			67
**Stroke Risk Scorecard**																																											
high risk	19	68%	9	32%	68.28	0.0000001	14	50%	14	50%	46.62	0.0000001	28	100%	0	0%	82.44	0.0000001	14	50%	14	50%	38.85	0.0000001	19	68%	9	32%	31.11	0.00000001	19	67.86%	9	32.14%	74.2	0.0000001	19	68%	9	32%	68.28	0.0000001	28
moderate risk	0	0%	29	100%			0	0%	29	100%			0	0%	29	100%			7	24%	22	76%			19	66%	10	34%			19	65.52%	10	34.48%			0	0%	29	100%			29
low risk	0	0%	59	100%			0	0%	59	100%			39	66%	20	34%			0	0%	59	100%			10	17%	49	83%			0	0.00%	59	100.00%			0	0%	59	100%			59
**Bathel Scale—assessment of the patient’s independence and fitness**																																											
complete dependency	14	54%	12	46%	28.94	0.00000001	10	38%	16	62%	18.2	0.0001	17	65%	9	35%	0.86	0.64	21	81%	5	19%	84.26	0.00000001	22	85%	4	15%	27.45	0.00001	21	80.77%	5	19.23%	34.34	0.00001	14	54%	12	46%	28.94	0.00001	26
partial independence	2	5%	35	95%			2	5%	35	95%			20	54%	17	46%			0	0%	37	100%			9	24%	28	76%			6	16.22%	31	83.78%			2	5%	35	95%			37
full independence	3	6%	50	94%			2	4%	51	96%			30	57%	23	43%			0	0%	53	100%			17	32%	36	68%			11	20.75%	42	79.25%			3	6%	50	94%			53
**Athens Insomnia Scale—one month after discharge**																																											
norm	0	0%	70	100%	51.89	0.0000001	0	0%	70	100%	33.11	0.0000001	32	46%	38	54%	14.93	0.00004	7	10%	63	90%	15.32	0.0004	12	17%	58	83%	52.2	0.000001	10	14.29%	60	85.71%	63.92	0.0000001	0	0%	70	100%	51.98	0.0000001	70
borderline of the norm	3	15%	17	85%			3	15%	17	85%			18	90%	2	10%			2	10%	18	90%			11	55%	9	45%			3	15.00%	17	85.00%			3	15%	17	85%			20
insomnia	16	62%	10	38%			11	42%	15	58%			17	65%	9	35%			12	46%	14	54%			25	96%	1	4%			25	96.15%	1	3.85%			16	62%	10	38%			26
Total	19	16%	97	84%			14	12%	102	88%			67	58%	49	42%			21	18%	95	82%			48	41%	68	59%			38	32.76%	78	67.24%			19	16%	97	84%			116

N—number; *p*—statistical significance; chi—chi square test.

**Table 5:** Distribution of survival 3 months in groups of dependent variables.

	Survival 3 Months						Total
	Yes		No		chi	*p*	N
	N	%	N	%			
**SGA—Subjective Global Assessment**							
well-nourished	40	83%	8	17%	30.28	0.000001	48
mildly/moderately malnourished	23	77%	7	23%			30
severely malnourished	11	29%	27	71%			38
**CHA_2_DS_2_-VA—assessment of the risk of thromboembolic complications in patients after surgery**							
1	43	73%	16	27%	4.32	0.04	59
≥2	31	54%	26	46%			57
**SARC-F—assessment of sarcopenia risk after discharge**							
low risk of sarcopenia	49	100%	0	0%	63.34	0.000001	49
high risk of sarcopenia	25	37%	42	63%			67
**Stroke Risk Scorecard**							
high risk	11	39%	17	61%	12.78	0.002	28
moderate risk	17	59%	12	41%			29
low risk	46	78%	13	22%			59
**Bathel Scale—assessment of the patient’s independence and fitness**							
complete dependency	10	38%	16	62%	9.65	0.008	26
partial independence	28	76%	9	24%			37
full independence	36	68%	17	32%			53
**Athens Insomnia Scale—one month after discharge**							
norm	55	79%	15	21%	17.95	0.0001	70
borderline of the norm	10	50%	10	50%			20
insomnia	9	35%	17	65%			26
Total	74	64%	42	36%			116

N—number; *p*—statistical significance; chi—chi square test.

**Table 6:** Comparison of the significance of mean grip force in groups of dependent variables.

	Day 2—Grip Strength					Day 5—Grip Strength					Day 7—Grip Strength					Day 10—Grip Strength				
	x	SD	F	*p*	η^2^	x	SD	F	*p*	η^2^	x	SD	F	*p*	η^2^	x	SD	F	*p*	η^2^
**SGA—Subjective Global Assessment**																				
well-nourished	25.21	6.49	2.02	0.14		26.85	7.00	4.49	0.01 A > C	0.07	28.95	8.00	3.69	0.03 A > C	0.06	30.42	9.29	1.23	0.30	
mildly/moderately malnourished	23.57	7.02				25.56	7.60				27.11	7.87				29.50	9.14			
severely malnourished	22.31	6.70				22.32	6.65				24.41	7.18				27.21	10.12			
**CHA_2_DS_2_-VA—assessment of the risk of thromboembolic complications in patients after surgery**																				
1	23.83	6.72	0.00	0.99		25.28	7.35	0.15	0.70		27.09	7.76	0.02	0.88		29.06	8.55	0.01	0.94	
≥2	23.84	6.85				24.77	7.23				26.87	8.08				29.20	10.57			
**SARC-F—assessment of sarcopenia risk after discharge**																				
low risk of sarcopenia	25.30	6.47	4.06	0.05 A > B	0.03	27.08	6.97	7.08	0.01 A > B	0.06	28.86	7.73	4.94	0.03 A > B	0.04	31.01	9.13	3.37	0.07	
high risk of sarcopenia	22.77	6.81				23.54	7.16				25.62	7.77				27.75	9.68			
**Stroke Risk Scorecard**																				
high risk	23.16	6.38	0.21	0.81		23.42	7.19	1.11	0.33		25.39	7.74	0.84	0.44		27.16	8.38	0.78	0.46	
moderate risk	23.80	7.13				24.83	7.04				27.02	7.62				29.78	10.46			
low risk	24.18	6.84				25.89	7.39				27.73	8.10				29.74	9.64			
**Bathel Scale—assessment of the patient’s independence and fitness**																				
complete dependency	24.07	6.78	0.15	0.86		22.94	7.04	1.53	0.22		25.26	8.03	1.31	0.27		27.56	10.32	0.50	0.61	
partial independence	23.34	6.43				25.17	7.01				26.49	7.39				29.18	9.85			
full independence	24.07	7.06				25.96	7.47				28.18	8.10				29.86	9.03			
**Athens Insomnia Scale—one month after discharge**																				
norm	25.48	6.42	5.70	0.004 A > B A > C	0.09	26.90	6.91	6.64	0.001 A > B A > C	0.10	29.00	7.69	6.57	0.002 A > B A > C	0.10	31.00	9.49	3.61	0.03 A > C	0.06
borderline of the norm	21.76	6.64				22.97	7.05				24.80	7.30				26.95	7.89			
insomnia	21.02	6.59				21.58	6.90				23.24	7.24				25.78	9.92			

*p*—statistical significance; x—average; SD—standard deviation; η^2^—measure of effect size.

**Table 7:** Comparison of the significance of the mean compressive force in groups of complications.

Complications	Day 2—Grip Strength					Day 5—Grip Strength					Day 7—Grip Strength					Day 10—Grip Strength				
	x	SD	F	*p*	η^2^	x	SD	F	*p*	η^2^	x	SD	F	*p*	η^2^	x	SD	F	*p*	η^2^
**Wound infection**																				
Yes	21.45	6.12	2.88	0.09		20.77	6.11	8.33	0.005	0.07	22.62	6.45	7.34	0.01	0.06	24.62	7.65	5.25	0.02	0.04
No	24.30	6.80				25.87	7.20				27.84	7.89				30.01	9.67			
**Tracheostomy**																				
Yes	21.69	6.24	1.61	0.21		21.05	5.87	4.95	0.03	0.04	22.91	6.42	4.38	0.04	0.05	24.99	7.68	3.04	0.08	
No	24.13	6.80				25.58	7.29				27.55	7.93				29.70	9.67			
**Dialysis after the procedure**																				
Yes	22.89	6.92	3.20	0.08		23.94	7.56	3.66	0.06		25.84	8.19	3.44	0.07		27.69	9.38	3.70	0.06	
No	25.14	6.36				26.52	6.62				28.56	7.24				31.10	9.53			
**Other complications**																				
Yes	23.12	6.59	0.28	0.60		21.43	6.19	6.62	0.01	0.05	23.75	7.02	4.45	0.04	0.04	26.35	10.08	2.20	0.14	
No	23.99	6.82				25.83	7.27				27.70	7.92				29.74	9.38			
**Delirium**																				
Yes	22.52	6.86	3.18	0.08		23.02	7.00	6.56	0.01	0.05	25.06	7.42	5.07	0.03	0.04	27.73	9.82	1.77	0.19	
No	24.77	6.57				26.45	7.16				28.35	7.97				30.12	9.30			
**Pressure ulcers**																				
Yes	22.31	6.70	2.93	0.09		22.32	6.65	8.39	0.005	0.07	24.41	7.18	6.33	0.01	0.05	27.21	10.12	2.30	0.13	
No	24.58	6.70				26.35	7.22				28.24	7.95				30.06	9.18			
**Sudden cardiac arrest**																				
Yes	21.45	6.12	2.88	0.09		20.77	6.11	8.33	0.005	0.07	22.62	6.45	7.34	0.01	0.06	24.62	7.65	5.25	0.02	0.04
No	24.30	6.80				25.87	7.20				27.84	7.89				30.01	9.67			
**Number of complications**																				
One	26.38	5.46	2.26	0.09		28.20	5.91	3.71	0.01 A > C A > D	0.09	30.06	6.71	2.78	0.04 A > D	0.06	31.92	7.92	1.36	0.26	
Two	23.49	7.14				25.06	7.80				27.00	8.69				28.69	10.14			
Three	21.87	7.51				24.17	7.87				25.96	8.07				28.63	10.30			
Four and more	23.07	6.37				22.05	6.17				24.29	6.89				26.97	9.54			

*p*—statistical significance; x—average; SD -standard deviation; η^2^—measure of effect size.

**Table 8:** Comparison of the significance of mean compressive strength in the 3-month survival groups.

	Day 2—Grip Strength					Day 5—Grip Strength					Day 7—Grip Strength					Day 10—Grip Strength				
	x	SD	F	*p*	η^2^	x	SD	F	*p*	η^2^	x	SD	F	*p*	η^2^	x	SD	F	*p*	η^2^
**3-month survival**																				
yes	24.86	6.63	4.85	0.03	0.05	26.36	7.25	7.27	0.01	0.06	28.18	8.05	4.86	0.03	0.04	30.37	9.70	3.53	0.06	
no	22.03	6.68				22.68	6.75				24.88	7.21				26.94	8.99			

*p*—statistical significance; x—average; SD -standard deviation; η^2^—measure of effect size.

**Table 9:** Key perioperative factors associated with the risk of developing sarcopenia.

Category	Parameters	Key Observations from the Study
**Mechanical ventilation time**	Number of minutes from intubation to extubation	Longer ventilation time (mean 762 min) was observed in patients with poorer general condition, which favored loss of muscle strength and delayed mobilization.
**Length of stay in ICU**	Number of hours from admission to discharge from intensive care	The stay in the ICU was very diverse (49–1321 h). Longer hospitalization was associated with a higher risk of complications, immobilization, and intensification of catabolic processes.
**Transfusions of blood and blood products**	FFP, PLCC, cryoprecipitate	In patients at high risk of sarcopenia and severe malnutrition, FFP, PLCC, and cryoprecipitate were used more frequently. In the severely malnourished group, transfusions were significantly more frequent (*p* = 0.000001).
**Postoperative complications**	Infections, delirium, dialysis, pressure ulcers, SCA, tracheostomy	Complications were more common in patients at high risk of sarcopenia and malnutrition. The text indicates that “wound infection, tracheostomy, bedside dialysis, delirium, bedsores, SCA, and other complications were significantly more common” in this group.
**Grip force dynamics**	Dynamometer measurements on the 1st and 2nd postoperative days	Grip strength was significantly higher in patients who survived for 3 months. The differences were particularly evident on the 2nd postoperative day and reached statistical significance

## Data Availability

The data presented in this study are available on request from the corresponding author, because it contains, among other things, sensitive data, which, in accordance with the provisions of the European Union RODO, require protection, including the protection of privacy.
